# Bibliometric analyses of social media for educational purposes over four decades

**DOI:** 10.3389/fpsyg.2022.1061989

**Published:** 2023-01-06

**Authors:** Zhonggen Yu, Paisan Sukjairungwattana, Wei Xu

**Affiliations:** ^1^Department of English Studies, Faculty of Foreign Studies, Beijing Language and Culture University, Beijing, China; ^2^Faculty of Liberal Arts, Mahidol University, Nakhon Pathom, Thailand; ^3^Faculty of Humanities and Social Sciences, City University of Macau, Taipa, Macau SAR, China

**Keywords:** bibliometric analysis, social media, education, educational outcomes, challenges

## Abstract

The unexpected outbreak of COVID-19 pandemic has led students to frequently use social media to receive education, which brought about both positive and negative learning outcomes (Oliveira et al., 2022). To address the issue of integrating social media into education, this study conducted both quantitative and qualitative studies using VOSviewer and CitNetExplorer. The qualitative study through CitNetExplorer, involving 1780 publications, concluded that while social media might have gained popularity in education based on the classic theoretical framework of the zone of proximal development, there might be many challenges such as teacher resistance, data privacy, costs, and ethical and social issues. Besides, this study conducted bibliometric analyses using VOSviewer (*N* = 1841) to identify the top cited authors, organizations, documents, references, sources, countries, and keywords with high occurrences based on the citation networks. In the future, researchers could enhance the studies on how to guide students and teachers to properly integrate social media into education.

## Introduction

The sudden outbreak of COVID-19 pandemic has led students to frequently use social media to implement distance learning, which brought about both positive and negative learning outcomes (Oliveira et al., 2022). Social media could expand the scope of social networks in users’ academic activities or their extracurricular education. However, teachers and parents were worried about the negative effects of excessive access to social media on children’s physical condition, personal information protection, and on-campus education interference (Lu, 2021). The frequent use of social media could positively influence digital reading comprehension although no positive influence was revealed on adolescent learners. The use of social media in eastern countries could not cause significant improvements in digital reading comprehension, while it could positively influence digital reading skills in western countries (Chen et al., 2021).

**Figure 1:**
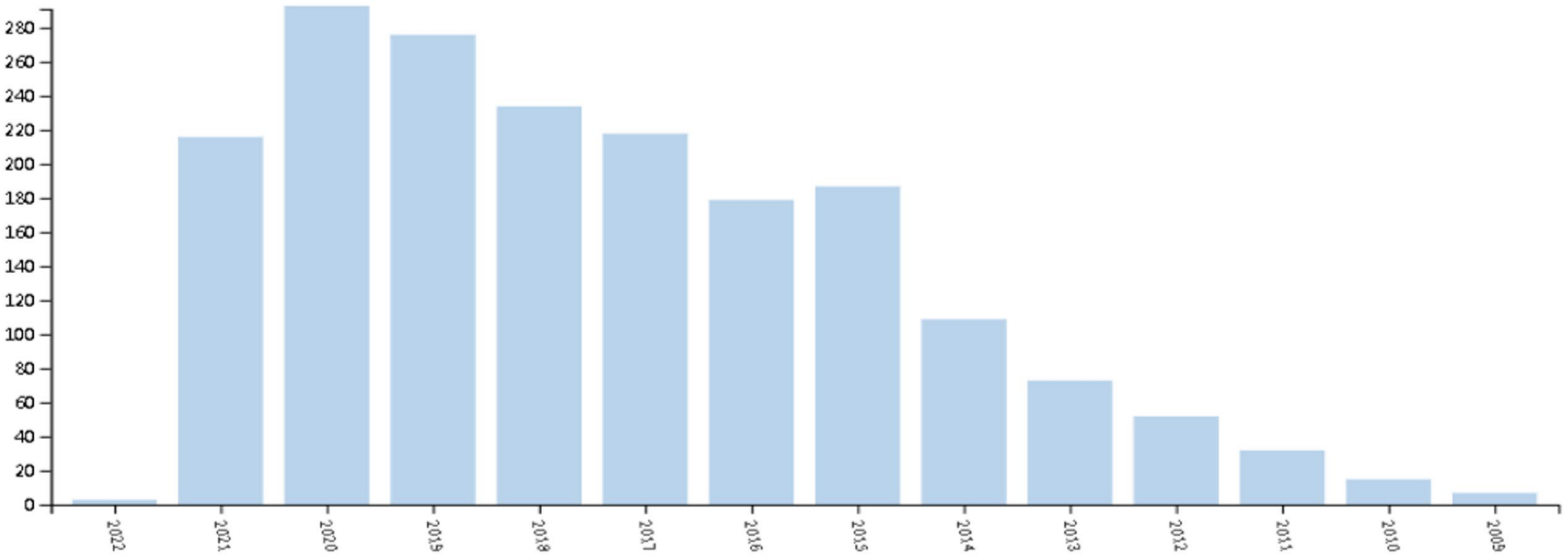
The research trend of the use of social media in education.

As shown in Figure 1, the use of social media for educational purposes has caught researchers’ attention since it was launched in the year 2009. The number of studies steadily went up until the year 2015 when the research reached a peak. After that, the number of relative studies fluctuated but with a steady growth until it arrived at the second peak in the year 2020. The year 2021 witnessed a slight decline in social media-assisted educational research possibly due to the delay in database inclusion. In general, the research into social media-assisted education has gained popularity in the recent decade. While the application of social media to education is increasingly drawing researchers’ attention with the development of information technologies, scanty studies have been devoted to the bibliometric analysis of the use of social media for the educational purpose (Barrot, 2021). This study, aiming to conduct bibliometric analyses of social media for educational purposes, seems meaningful since it attempts to complement the scanty studies by combining VOSviewer with CitNetExplorer.

## Theoretical framework

Social constructivism theory has provided a theoretical foundation for the use of social media in education. Social constructivism theory assumes that social connections with peers, teachers, or friends play an important role in educational outcomes (Manca, 2020). This theory is in essence student-centered, where social interactions are deemed as a key factor that facilitates learning since students can share opinions, solve problems, and collaborate through mutual communication. The frequent social interactions assisted with social media tools could increase students’ access to knowledge and enhance their motivation to learn. The established social networks might provide a sea of learning resources where students could receive new information and insights.

Interdisciplinary collaboration is becoming increasingly important in the contemporary and future research since scientific issues are growing as systematic problems in need of collaboration of experts from various disciplines (Domik and Fischer, 2011, p. 129). Interdisciplinary experts can work collaboratively through social platforms or any learning management system to work on the same scientific issue in the real-world laboratory (Lu, 2021). Learners and teachers can also deliver and absorb knowledge through multiple platforms and close social networks based on social media or educational platforms. It is thus necessary to analyze the effects of social media on educational outcomes. Bibliometric analyses can provide solid references for the relevant research by clustering research publications and shedding light on top authors, organizations, countries, keywords, documents, the sources. This study aims to identify the effects of social media on educational outcomes through a bibliometric analysis.

## Literature review

Numerous social media have been widely used in education and exerted a great influence on educational outcomes. Facebook was found able to improve learning outcomes while no gender differences were found. Twitter could also improve educational outcomes in most countries. However, it also made the distinctions unclear between education, entertainment, and social interactions (Manca, 2020), which might have negatively influenced the effectiveness of educational practice. Given previous inconsistent findings, we attempted to examine the effect of social media on educational outcomes using CitNetExplorer. We, therefore, proposed the following research question:

Can the Use of social media improve educational outcomes?

Given the important role of bibliometric analysis in the advancement of educational studies, numerous studies adopted this method to explore related areas, e.g., the use of technology (Shen and Ho, 2020), the management of education (Hallinger and Kovacevic, 2019), education of medical sciences (Azer, 2015), and linguistic science education (Barrot, 2020). Very few studies examined the use of social media in education *via* a meta-analysis although some studies explored the applications of social media using critically analytical (e.g., Sterling et al., 2017; Nagle, 2018; Bruguera et al., 2019) and meta-analytical methods (e.g., Marker et al., 2018). Most of these meta-analyses and critical analyses focused on the technological issue of social media platforms.

While seldom studies combined VOSviewer with CitNetExplorer to conduct bibliometric analyses, several studies analyzed the use of social media in education through bibliometric analyses or meta-analyses. Through a bibliometric analysis, Facebook was found to be widely accepted by learners in the United States, Australia, Turkey, the United Kingdom, and Taiwan and “Computers and Education” was the most influential journal to publish the research into social media for educational purposes. The studies on the use of social media in education could be integrated into other disciplines such as computing technologies, language sciences, and medical sciences (Lopes et al., 2017). WhatsApp was considered a popular social media for educational purposes while Facebook and Twitter outweighed other social media (Manca, 2020). Considering the scanty studies on the bibliometric analyses of social media for educational purposes, we tried to conduct bibliometric analyses using VOSviewer. This study proposed the second research question as follows:

What are the top authors, organizations, countries, keywords, documents, sources, and references in social media-assisted educational research?

## Materials and methods

To answer the first research question, we conducted a qualitative analysis using CitNetExplorer. On November 19, 2022, we searched the Core Collection of Web of Science by entering “social media” and educat* OR learn* OR teach* as titles, leading to 2015 results. We entered the result into Endnote 20 to check and remove duplications. The specific process was click “library” and “find duplicates” in the command column. Then the program would compare and find duplicates. We then selected the records to keep. The records not selected would be moved to trash.

**Figure 2:**
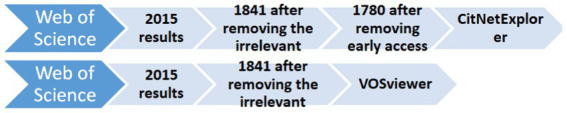
A flow chart of the research procedure.

Two independent researchers perused the results and removed those unrelated, leading to the final 1,841 results (Figure 2). They adopted a four-step approach to making a decision. Firstly, they read the titles to determine if they were relevant to this research. They would remove them if they found them irrelevant. Otherwise, they would continue to read the abstract to determine the relevance as a second step. Thirdly, if titles and abstracts were relevant, they would read the conclusion to decide on the relevance. Fourthly, if both of them failed to reach an agreement on any decision, a third rater would be invited to make the final decision. The inter-rater reliability (*k* = 0.84) reached a satisfactory level. We removed the document type “early access” to avoid the system errors. The final included documents (*N* = 1780) included articles (*N* = 941), proceedings papers (*N* = 586), meeting abstracts (*N* = 121), editorial materials (*N* = 84), early access (*N* = 61), review articles (*N* = 60), letters (*N* = 34), book reviews (*N* = 22), news items (*N* = 5), and book chapters (*N* = 4). The classification might be overlapped and the total number might be thus not equal to the total results.

To answer the second research question, we conducted a bibliometric analysis using VOSviewer. We used the obtained results (*N* = 1841) without removing the “early access” documents since no systematic errors occurred (Figure 2). We implemented the bibliometric analyses *via* co-authorship (authors, organizations, and countries), co-occurrence (all keywords, author keywords, and keywords plus), citation (documents, sources, authors, organizations, and countries), bibliographic coupling (documents, sources, authors, organizations, and countries), and co-citation (cited references, cited sources, and cited authors).

## Results

This result section aims to analyze the scattering of highly cited publications for the use of social media in educational fields based on Bradford’s law (Venable et al., 2016). It highlights the most highly cited authors, organizations, countries, keywords, documents, sources, and references, as well as the educational outcomes due to the use of social media.

Can the use of social media improve educational outcomes?

**Figure 3:**
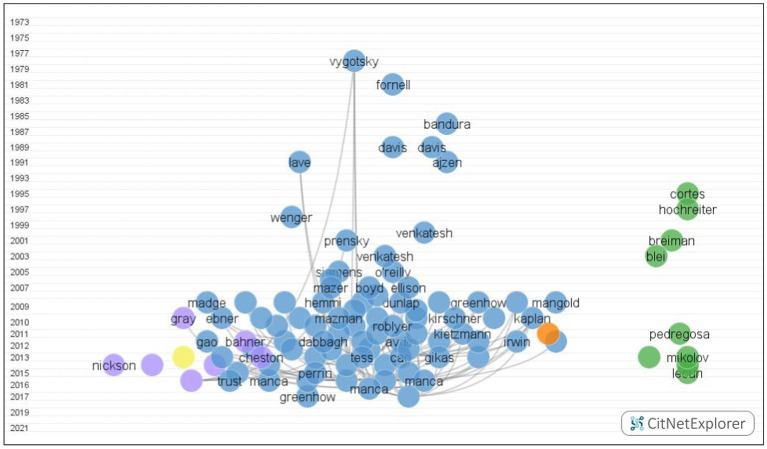
Citation networks of 100 publications.

The networks constructed by CitNetExplorer consisted of 1,967 publications ranging from 1978 to 2021, and 4,929 citation links. The reason why the publications outnumber the originally retrieved literature is that the citation network includes both predecessors and successors. Predecessors refer to the publications cited by a certain number of publications in the current network, while successors indicate those citing a certain number of publications in the current network (Van Eck and Waltman, 2017). Clustering techniques in CitNetExplorer divided the citation networks into six groups by merging small clusters (Figure 3).

Table 1 presents detailed data regarding several variables, which could provide a meaningful reference for readers, researchers, and practitioners. The minimum cluster size was set at 10, and the resolution was set at 1. Some publications (*N* = 644) did not belong to any cluster.

**Table 1:** Six clusters and citation networks.

Cluster	Color	No. of publications	No. of citation link	No. of publications ≧10 citations	No. of publications in 100 most cited publications
1	Blue	796	3,571	156	82
2	Green	237	451	24	9
3	Purple	189	475	20	7
4	Orange	63	95	7	1
5	Yellow	22	23	2	1
6	Brown	16	16	2	0

We focused our exploration on the first cluster since it presented the largest number of publications, citation links, the most publications with more than 10 citations, and the top number of documents in 100 most cited publications (Table 1). We collected data from pioneering publications, four studies with top citation scores, and the most recent studies to generalize the major topics and discuss the implications (Table 2).

**Table 2:** Major findings *via* the first cluster.

Item	Year	Citation	1st author	Title	Source
Pioneer	1978	44	Vygotsky	Mind in society: Development of higher psychological processes	Harvard University Press
Highest citation score	2010	154	Kaplan	Users of the world, unite! The challenges and opportunities of Social Media	Business Horizons
Second citation score	2013	93	Tess	The role of social media in higher education class (real and virtual)-a literature review	Computers in Human Behavior
Third citation score	2007	78	Boyd	Social network sites: Definition, history, and scholarship	Journal of computer-mediated communication
Fourth citation score	2010	64	Roblyer	Findings on Facebook in higher education: A comparison of college faculty and student uses and perceptions of social networking sites	Internet and Higher Education
Most recent	2021	1	Marin	Pre-service teachers’ perceptions of social media data privacy policies	British Journal of Educational Technology
Topic of discussion	Zone of proximal development; benefits and challenges of social media; data privacy policy				
Overall conclusion	The theory of the zone of proximal development may be connected to the use of social media in education despite the concern of data privacy				

As shown in Table 2, much earlier, the study authored by Vygotsky (1978), with the highest citation score, paved a solid theoretical foundation for the use of social media in education. Previously, it was generally maintained that the learning process and development of human brain coincided with each other. However, Vygotsky (1978) altered it by arguing that the learning process could create the zone of proximal development and that the learning process preceded the zone of proximal development. The use of social media in learning might be able to improve the zone of proximal development by way of the enhancement of interactions between users, peers, or superiors. This process seems like the argument that “good learning” could improve the zone of proximal development *via* the interactions between children and adults (Vygotsky, 1978), leading to improved learning outcomes.

Social media could be conceptualized under the theoretical framework where social presence, media richness, self-presentation, and self-disclosure were involved (Kaplan and Haenlein, 2010). The theory of social presence (Short et al., 1976) indicated the aural, oral, visual, or physical contacts realized during the communication process through social media. Social presence was positively correlated with social influence. The theory of media richness (Daft and Lengel, 1986) referred to the assumption that social media might resolve ambiguity and reduce vagueness. The notion “richness” indicated the amount of information transmitted through social media which was positively related to the ability to reduce ambiguity and vagueness. The theory of social presentation meant that people desired to influence others during the social media-assisted communication. They might either wish to obtain rewards or establish their own social identities or statuses (Bin Tareaf et al., 2020), which could be realized through self-disclosure. Self-disclosure acted as an important step for communicators to establish mutually trustable interpersonal relationships.

Characterized by social presence, media richness, self-presentation, and self-disclosure, social media might improve the zone of proximal development when used in education. Social presence could increase the frequency of contacts by way of various kinds of interactions, which might be conducive to the zone of proximal development in human brains. The increasing amount of information could also help communicators resolve misunderstandings and misconceptions, which could improve learning effectiveness and facilitate the proximal development. Through self-disclosure, learners could make every effort to enhance their self-representation and influence others by enhancing their learning identities. This might enhance their self-efficacy and improve their interactions with cyber friends or learning peers, which encouraged students to adopt social media in their learning process.

Surprisingly, the study with the fourth citation score (Roblyer et al., 2010) found that students and teachers held contradictory attitudes toward social media. Social media, providing rich learning resources for learners and teachers, have been widely accepted by students in education. Students tended to be more open to innovative technologies and readier to accept them such as social media than teachers in the educational process. However, teachers were more likely to implement the teaching practice using traditional methods, e.g., email (Roblyer et al., 2010). Pharmacy majors held positive attitudes toward the use of social media in learning although they felt uneasy when confronted with teachers. Teachers, who had no enough social media-assisted teaching experience, were not accustomed to the use of social media in their teaching practice (Mawdsley, 2015). Teachers might resist changing their traditional and convenient methods since the alteration was in need of many efforts in both time and energy. Data privacy might also be a serious issue that they, as adults, were aware of.

Social media has gained popularity applied to the field of education, where data privacy has drawn much attention. Pre-service teachers, who were aware of both benefits and challenges of educational social media, held neutral attitudes toward the use of social media for educational purposes. Their attitudes toward the educational social media were not proven related to the awareness of data privacy (Marín et al., 2021). This paradoxical finding was consistent with a previous study (Obar and Oeldorf-Hirsch, 2020), which indicated that although users of social media tended to claim that they attached much importance to their privacy when using social media, they ignored this when they were requested to answer the question digitally. Similarly, educators tended to disregard the issue of data privacy when they ticked the question such as “do you agree to the term?” (Walster, 2017).

Contradictory findings were revealed regarding the concern with data privacy in the use of social media. While numerous researchers concluded that data privacy might be a serious issue users worry about, Walster (2017) revealed that educators might not be concerned with the issue of data privacy when they applied social media to education despite their strong awareness of privacy disclosure. The majority of medical learners showed their worries about their privacy disclosure when using social media (Flickinger et al., 2015). They, therefore, changed their privacy settings to prevent possible revelation of personal information (Walton et al., 2015). In addition to data privacy, some learners were worried about their poor digital literacy (O’Connor et al., 2021), which might hinder their effective use of social media in education.

The study with the second citation score proposed some problems regarding the use of social media in higher education (Tess, 2013). The infrastructure for the use of social media might be costly, which was hard to be afforded in some countries or areas. The effectiveness of social media used in higher education was still dependent since researchers had just begun to collect relevant data. Teachers, who preferred traditional teaching tools, might resist adopting social media in education. Those who accepted social media in education might hold suspicious attitudes because they believed it was merely at the beginning stage (Galan et al., 2015), so was the application of other educational technologies (Tess, 2013). While social media were becoming increasingly popular on campus, the viability of their use in education had not been comprehensively demonstrated. The social media use in education is at a testing stage at an individual rather than an institutional level.

The study with the third citation score is focused on various sites of social media (Boyd and Ellison, 2007). Various social media shared in common their maintenance of social connections. Some of them aimed to cluster users and cater to their various needs based on their different preferences such as political views, cultures, habits, economic statuses, genders, or educational levels. Social media also varied in terms of the amount of updated information, communicative platforms, opinion sharing, and mobile connectivity. The variety of social media might have caused the difficulty for students to gain easy access to each of them. Different preferences and digital literacy between students and teachers might have weakened their motivation to learn or teach assisted with social media.

However, researchers did attempt to connect social media to educational institutions, e.g., schools, colleges, universities, and libraries. Students’ perceptions were revealed about the presence of teachers in the network built by Facebook (Ellison et al., 2007), which could influence teacher-student relationships (Mazer et al., 2007). Librarians held positive attitudes toward the use of social media in a library and suggested allowing minors to have free access to social media in the library (Charnigo and Barnett-Ellis, 2007). While there might be benefits of the use of social media in education, scanty studies had been conducted on the effect of social media on educational outcomes. Bibliometric analyses were supposed to complement this regret.

What are the top authors, organizations, countries, keywords, documents, sources, and references in social media-assisted educational research?

VOSviewer could construct the citation networks, conducive to bibliometric analyses. VOSviewer, complementing the functions of CitNetExplorer, aims to construct and visualize bibliometric networks at a collective level, while CiNetExplorer attempts to cluster the publications at an individual level (Van Eck and Waltman, 2017). We analyzed the co-authorship, co-occurrence, citation, bibliographic coupling, and co-citation using VOSviewer to complement the qualitative analyses using CitNetExplorer.

We conducted the bibliometric analysis based on the obtained data (*N* = 1967). VOSviewer read bibliometric data files collected from Web of Science. By choosing co-authorship as the analysis type and authors, organizations, and countries as analysis units, we obtained the top 10 authors, organizations, and countries based on the citations (Table 3).

**Table 3:** Top 10 authors, organizations, and countries based on the citations.

*N*	Author	Document	Citation	Total link strength	*N*	Author	Document	Citation	Total link strength
1	Manca, Stefania	8	322	4	6	Popescu, Elvira	5	78	0
2	Greenhow, Chirstian	13	272	18	7	Haythornthwaite, Caroline	5	32	6
3	Al-rami, Waleed Mugahed	7	212	0	8	Capenter, Jeffrey, P.	6	29	0
4	Balakrishnan, Vimala	6	143	0	9	Brandon, Diana, L.	5	20	14
5	Sherbino, Jonathan	6	101	0	10	Oliveira, Luciana	5	15	0
***N***	**Organization**	**Document**	**Citation**	**Total link strength**	***N***	**Organization**	**Document**	**Citation**	**Total link strength**
1	George Mason University	7	699	6	6	Boston University	5	331	8
2	University of Minnesota	6	503	5	7	National Research Council of Italy	7	320	4
3	John Hopkins University	11	454	9	8	The University of Hongkong	19	319	8
4	Harvard University	7	379	4	9	University of California-Los Angeles	12	269	8
5	Michigan State University	26	333	23	10	University of Malaya	13	263	0
***N***	**Country**	**Document**	**Citation**	**Total link strength**	***N***	**Country**	**Document**	**Citation**	**Total link strength**
1	United States	529	7,066	185	6	Spain	81	704	77
2	England	172	1,430	121	7	Malaysia	67	616	53
3	China	130	1,121	90	8	Germany	53	375	66
4	Australia	121	950	105	9	Italy	31	368	21
5	Canada	84	924	70	10	Saudi Arabia	65	359	56

By choosing co-occurrence as the analysis type and all keywords, author keywords, and keywords plus as the analysis units respectively, we obtained the top 30 keywords according to the sequence of the number of occurrences (Table 4).

**Table 4:** Top 10 keywords according to occurrences.

*N*	All keywords	Occurrence	Total link strength	*N*	All keywords	Occurrence	Total link strength
1	Social media	903	3,084	6	Education	142	546
2	Facebook	239	1,349	7	Higher education	107	529
3	Twitter	172	844	8	Perceptions	64	477
4	Technology	123	670	9	Communication	71	386
5	Students	101	575	10	Networking	60	372
***N***	**Author keywords**	**Occurrence**	**Total link strength**	***N***	**Author keywords**	**Occurrence**	**Total link strength**
1	Social media	903	1,296	6	Deep learning	75	139
2	Higher education	107	236	7	Twitter	71	183
3	Machine learning	94	188	8	E-learning	49	95
4	Education	83	165	9	Sentiment analysis	41	97
5	Facebook	80	186	10	Learning	36	96
***N***	**Keywords plus**	**Occurrence**	**Total link strength**	***N***	**Keywords plus**	**Occurrence**	**Total link strength**
1	Facebook	178	699	6	Perceptions	63	311
2	Twitter	120	379	7	Education	61	173
3	Technology	99	359	8	Networking	59	243
4	Students	87	302	9	Online	58	212
5	Information	65	221	10	Communication	56	228

We obtained the top 10 documents, sources, authors, organizations, and countries according to the rank of citations by choosing citation as the analysis type and documents, sources, authors, organizations, and countries as analysis units (Table 5).

**Table 5:** Top 10 documents, sources, authors, organizations, and countries based on citations.

*N*	Document	Citation	Link	*N*	Document	Citation	Link
1	Dabbagh and Kitsantas (2012)	662	63	6	Bode (2016)	175	9
2	Gikas and Grant (2013)	510	30	7	Attai et al. (2015)	166	2
3	Tess (2013)	416	99	8	Greenhow et al. (2016)	161	28
4	Witherspoon et al. (2013)	303	58	9	Manca (2020)	157	45
5	Kaplan and Haenlein (2016)	210	6	10	Leonardi (2015)	150	9
***N***	**Source**	**Document**	**Citation**	***N***	**Source**	**Document**	**Citation**
1	Internet and Higher education	9	1,451	6	Sustainability	16	156
2	Computers in Human Behavior	16	1,009	7	International Review of Research in Open and Distributed Learning	9	140
3	Journal of Medical Internet Research	13	292	8	Journal of Marketing for Higher Education	6	124
4	Learning Media and Technology	5	247	9	Education and Information Technologies	8	122
5	British Journal of Educational Technology	9	243	10	Nurse Education Today	7	109
***N***	**Author**	**Document**	**Citation**	***N***	**Author**	**Document**	**Citation**
1	Manca, Stefania	8	322	6	Popescue, Elvira	5	78
2	Greenhow, Christine	13	272	7	Haythornthwaite, Caroline	5	32
3	Al-rami, Waleed Mugahed	7	212	8	Carpenta, Jeffery P.	6	29
4	Balakrishnan, vimala	6	143	9	Brandon, Diana, L.	5	20
5	Sherbino, Jonathan	6	101	10	Oliveira, Luciana	5	15
***N***	**Organization**	**Document**	**Citation**	***N***	**Organization**	**Document**	**Citation**
1	George Manson University	7	699	6	Boston University	5	331
2	University of Minnesota	6	503	7	National Research Council of Italy	7	320
3	Johns Hopkins University	11	454	8	University of Hongkong	19	319
4	Harvard University	7	379	9	University of California-Los Angeles	12	269
5	Michigan State University	26	333	10	University of Malaya	13	263
***N***	**Country**	**Document**	**Citation**	***N***	**Country**	**Document**	**Citation**
1	United States	529	7,066	6	Spain	81	704
2	England	172	1,430	7	Malaysia	67	616
3	China	130	1,121	8	Germany	53	375
4	Australia	121	950	9	Italy	31	368
5	Canada	84	924	10	Saudi Arabia	65	359

By choosing bibliographic coupling as the analysis type and documents, sources, authors, organizations, and countries as analysis units, we obtained the top 10 documents, sources, authors, organizations, and countries based on citations, respectively (Table 6).

**Table 6:** Top 10 documents, sources, authors, organizations, and countries based on citations.

*N*	Document	Citation	Link	*N*	Document	Citation	Link
1	Dabbagh and Kitsantas (2012)	662	106	6	Bode (2016)	175	78
2	Gikas and Grant (2013)	510	307	7	Greenhow et al. (2016)	166	2
3	Tess (2013)	416	1,043	8	Manca (2020)	161	652
4	Witherspoon et al. (2013)	303	125	9	Leonardi (2015)	157	643
5	Kaplan and Haenlein (2016)	210	168	10	De Zuniga et al. (2017)	150	158
***N***	**Source**	**Document**	**Citation**	***N***	**Source**	**Document**	**Citation**
1	Internet and higher education	9	1,451	6	Sustainability	16	156
2	Computers in Human Behavior	16	1,009	7	International Review of Research in Open and Distributed Learning	9	140
3	Journal of Medical Internet Research	13	292	8	Journal of Marketing for Higher Education	6	124
4	Learning Media and Technology	5	247	9	Education and Information Technologies	8	122
5	British Journal of Educational Technology	9	243	10	Nurse Education Today	7	109
***N***	**Author**	**Document**	**Citation**	***N***	**Author**	**Document**	**Citation**
1	Manca, Stefania	8	322	6	Popescue, Elvira	5	78
2	Greenhow, Christine	13	272	7	Haythornthwaite, Caroline	5	32
3	Al-rami, Waleed Mugahed	7	212	8	Carpenta, Jeffery P.	6	29
4	Balakrishnan, vimala	6	143	9	Brandon, Diana, L.	5	20
5	Sherbino, Jonathan	6	101	10	Oliveira, Luciana	5	15
***N***	**Organization**	**Document**	**Citation**	***N***	**Organization**	**Document**	**Citation**
1	George Manson University	7	699	6	Boston University	5	331
2	University of Minnesota	6	503	7	National Research Council of Italy	7	320
3	Johns Hopkins University	11	454	8	University of Hongkong	19	319
4	Harvard University	7	379	9	University of California-Los Angeles	12	269
5	Michigan State University	26	333	10	University of Malaya	13	263
***N***	**Country**	**Document**	**Citation**	***N***	**Country**	**Document**	**Citation**
1	United States	529	7,066	6	Spain	81	704
2	England	172	1,430	7	Malaysia	67	616
3	China	130	1,121	8	Germany	53	375
4	Australia	121	950	9	Italy	31	368
5	Canada	84	924	10	Saudi Arabia	65	359

By choosing co-citation as the analysis type and cited references, cited sources, and cited authors as the analysis units, we obtained the top 10 cited references, cited sources, and cited authors according to the number of citations (Table 7).

**Table 7:** Top 10 cited references, cited sources, and cited authors based on citations.

*N*	Cited references	Citation	Link	*N*	Cited references	Citation	Link
1	Kaplan and Haenlein (2010)	159	471	6	Junco et al. (2011)	62	349
2	Tess (2013)	98	470	7	Witherspoon et al. (2013)	58	74
3	Boyd and Ellison (2007)	79	248	8	Moran et al. (2011)	49	197
4	Roblyer et al. (2010)	65	390	9	Kirschner and Karpinski (2010)	48	345
5	Dabbagh and Kitsantas (2012)	63	263	10	Davis (1989)	46	230
***N***	**Cited Source**	**Citation**	**Link**	***N***	**Cited Source**	**Citation**	**Link**
1	Computers in Human Behavior	1,180	35,999	6	Journal of Computer Assisted Learning	326	8,320
2	Computers and Education	912	25,701	7	Mis Quarterly	323	11,641
3	Internet and Higher Education	525	13,494	8	Journal of Computer-mediated Communication	305	7,680
4	British Journal of Educational Technology	399	10,769	9	Business Horizons	299	6,473
5	Journal of Medical Internet Research	352	4,530	10	Learning, Media and Technology	239	6,491
***N***	**Cited Author**	**Citation**	**Link**	***N***	**Cited Author**	**Citation**	**Link**
1	Greenhow, C	222	2,887	6	Venkatesh, V	99	1,243
2	Kaplan, AM	194	1,333	7	Tess, PA	99	1,082
3	Junco, R	193	2,107	8	Veletsianos, G	93	1,182
4	Manca, S	165	2,119	9	Carpenter, JP	93	940
5	Selwyn, N	144	1,577	10	Al-rahmi, WM	86	1,239

## Discussion

Social media-assisted educational effectiveness might be improved under the theoretical framework of the zone of proximal development despite concerns with numerous challenges. Pre-service teachers considered social media beneficial tools to encourage students to raise questions, adopt collaborative learning, and share opinions by bridging the gap between social media and their zones of proximal development (Alghamdi and Alanazi, 2019). The socio-cultural theory supported the use of social media for educational purposes, where learners’ self-regulation, the zone of proximal development, and scaffolding education were involved (Zhang et al., 2013; Cappellini, 2016). The asynchronous and synchronous social media-assisted pedagogical approaches could provide students with comprehensive scaffolding learning resources and cultivate a harmonious learning environment, leading to slight improvements in German language proficiency (Dobberfuhl-Quinlan, 2018).

However, inconsistent findings were revealed regarding the use of social media for educational purposes. Although social media might be able to facilitate learning due to their interactive and connective features, teachers might prefer traditional pedagogies to those integrated with social media. Teachers and students might evaluate the use of social media in education differently or even contradictorily. Teachers with lower digital literacy might be resistant to the acquisition of new applications in their traditional teaching process. Teachers might worry about the affordances of digital technologies and the uncertainty of contents in social media. The use of social media could by no means guarantee successful teaching or learning (Forbes, 2017).

The use of social media in education might bring about challenges although it could improve educational outcomes. The business models of social media made it easy to reveal personal information. Challenges existed in the use of social media in education, where teachers’ online profiles might be exposed to risky virtual communities (Forbes, 2017). Personal data privacy is one of the major concerns in the use of social media for educational purposes. Knowledge about the data revelation was not available to educators and students, who might be unaware of their revelation of privacy (Marín et al., 2022). However, another study found that many students could avoid the privacy revelation by easily modulating the settings in social media (Walton et al., 2015). The paradoxical findings may highlight the importance of data privacy in the use of social media for educational purposes.

Worse, the use of social media in education could never guarantee the ethical or social commitments. Teachers or students might be indulged in entertainment rather than education on their social media due to the absence of strong awareness of educational benefits (Willems et al., 2018). The fast development of social media led to many disputes about whether they could be used in higher education. Many universities and colleges took cautious measures to deal with the challenges and benefits that might be caused by the use of social media (Au et al., 2015). Although technologies such as social media could quickly fix educational problems, this quick fix might bring about in-depth and unexpected educational issues (Selwyn, 2014), including social and ethical concerns.

The construction of the infrastructure may also pose a threat to the use of social media for educational purposes. The expenditure of construction of the costly infrastructures might have also hindered the attempt to use social media for educational purposes. Learners could suffer from the unstable Internet connection, security problems, and costly purchase of equipment, which formed barriers to voluntary use of social media in education (Wickramanayake and Jika, 2018). The frequent use of technologies such as social media could lead to an engaged lifestyle, various social or ethical responsibilities, and quick working rhythms, which could make learners shoulder heavy burdens (Weatherspoon et al., 2015). While users could have inexpensive access to various kinds of social media, the frequent access could be accumulated to high living costs and heavy working burdens.

The bibliometric analysis of the use of social media for educational purposes could provide references for researchers and practitioners. When they would like to design the applications of social media for educational purposes, they could consult the top authors, e.g., Manca Stefania, Greenhow Chirstian, Al-rami Waleed Mugahed, Balakrishnan Vimala, and Sherbino Jonathan. They could visit the highly cited organizations such as George Mason University, University of Minnesota, John Hopkins University, Harvard University, and Michigan State University. They could pay much attention to the top countries that examined the use of social media for educational purposes, e.g., the United States, England, China, Australia, and Canada. They could peruse the publications in the top journals, e.g., Internet and Higher Education, Computers in Human Behavior, Journal of Medical Internet Research, Learning Media and Technology, and British Journal of Educational Technology. Among the various social media, Facebook and Twitter were the kinds most frequently mentioned. To provide a reliable reference for future research, it is necessary to conduct bibliometric analyses to facilitate the integration of social media into education.

## Conclusion

### Major findings

This study conducted both quantitative and qualitative studies into the use of social media in education using VOSviewer and CitNetExplorer. The qualitative study through CitNetExplorer concluded that while social media might have gained popularity in education based on the classic theoretical framework of the zone of proximal development, there might be many challenges such as teacher resistance, data privacy, costs, and ethical and social issues. In this regard, much needs to be done to improve the effective integration of social media into education. Besides, this study conducted bibliometric analyses using VOSviewer to identify the top cited authors, organizations, documents, references, sources, countries, and keywords with high occurrences based on the citation networks. Researchers and practitioners can seek the literature according to the findings, which may facilitate their research and practice effectiveness.

### Limitations

The generalizability of the findings is subject to certain limitations. This study might have missed some literature due to the limitation of library sources. Databases other than Web of Science were not included in this study, which might have caused publication bias. The citation networks that guide this study may fail to represent all the research themes in the use of social media for educational purposes. Researchers may need to include a wider scope of literature to examine the use of social media for educational purposes.

### Future research directions

In the future, researchers could enhance the studies on how to guide students and teachers to properly integrate social media into education. Various kinds of social media may have puzzled users who may feel it hard to decide which social media they should choose. How to leverage the features of social media to improve learning outcomes may be meaningful for future research. The use of social media for educational purposes might not have been widely accepted by educational institutions although a growing number of students are prone to this new learning method. Future researchers can study how to encourage teachers to use social media, which may, in turn, improve the acceptance of educational institutions. Future advanced machine-learning technologies could also be integrated into social media design to power the educational construction of social networks (Lecun et al., 2015).

The pandemic has greatly increased the frequency of access to social media to share opinions, concerns, and experiences, where people could collaborate in learning to address the educational issue during the lockdown (Johnstone and Towbin, 2022). The COVID-19 pandemic requires the strategies of use of social media in medical fields to contain the pandemic (Bora et al., 1800). Future researchers could thus figure out how to improve the online, remote, or blended educational outcomes using social media, especially during the unpredictable pandemic time.

## Data availability statement

The original contributions presented in the study are included in the article/supplementary material, further inquiries can be directed to the corresponding author.

## Author contributions

ZY conceptualized, designed, collected, analyzed the data, wrote, edited, and polished this article. PS and WX revised, proofed, funded and edited the article. All authors contributed to the article and approved the submitted version.

## Funding

This work was supported by 2019 MOOC of Beijing Language and Culture University (MOOC201902) (Important) “Introduction to Linguistics”; “Introduction to Linguistics” of online and offline mixed courses in Beijing Language and Culture University in 2020; Special fund of Beijing Co-construction Project-Research and Reform of the “Undergraduate Teaching Reform and Innovation Project” of Beijing Higher Education in 2020-Innovative “Multilingual +” Excellent Talent Training System (202010032003); The research project of Graduate Students of Beijing Language and Culture University “XJ: The Governance of China” (SJTS202108).

## Conflict of interest

The author declares that the research was conducted in the absence of any commercial or financial relationships that could be construed as a potential conflict of interest.

## Publisher’s note

All claims expressed in this article are solely those of the authors and do not necessarily represent those of their affiliated organizations, or those of the publisher, the editors and the reviewers. Any product that may be evaluated in this article, or claim that may be made by its manufacturer, is not guaranteed or endorsed by the publisher.
